# Transcriptome Reveals 1400-Fold Upregulation of APOA4-APOC3 and 1100-Fold Downregulation of GIF in the Patients with Polycythemia-Induced Gastric Injury

**DOI:** 10.1371/journal.pone.0140534

**Published:** 2015-10-20

**Authors:** Kang Li, Luobu Gesang, Zeng Dan, Lamu Gusang, Ciren Dawa, Yuqiang Nie

**Affiliations:** 1 High altitude Medical Research Institute, People’s Hospital of Tibet Autonomous Region, Lhasa, 850000, China; 2 Department of Gastroenterology, People’s Hospital of Tibet Autonomous Region, Lhasa, 850000, China; 3 Department of Cardiology, People’s Hospital of Tibet Autonomous Region, Lhasa, 850000, China; 4 Department of Gastroenterology, Guangzhou First People’s Hospital, Guangzhou Medical University, Guangzhou, 510180, China; The University of Texas MD Anderson Cancer Center, UNITED STATES

## Abstract

High-altitude polycythemia (HAPC) inducing gastric mucosal lesion (GML) is still out of control and molecular mechanisms remain widely unknown. To address the issues, endoscopy and histopathological analyses were performed. Meanwhile, microarray-based transcriptome profiling was conducted in the gastric mucosa from 3 pairs of healthy subjects and HAPC-induced GML patients. HAPC caused morphological changes and pathological damages of the gastric mucosa of GML patients. A total of 10304 differentially expressed genes (DEGs) were identified, including 4941 up-regulated and 5363 down-regulated DEGs in gastric mucosa of GML patients compared with healthy controls (fold change ≥2, P<0.01 and FDR <0.01). Particularly, apolipoprotein genes APOA4 and APOC3 were 1473-fold and 1468-fold up-regulated in GML patients compared with the controls. In contrast, gastric intrinsic factor (GIF) was 1102-fold down-regulated in GML patients compared with the controls. APOA4 (chr11:116691770–116691711), APOC3 (chr11:116703530–116703589) and GIF (chr11:59603362–59603303) genes are all located on chromosome 11. APOA4 and APOC3 act as an inhibitor of gastric acid secretion while gastric acid promotes ulceration. GIF deficiency activates a program of acute anemia, which may antagonize polycythemia while polycythemia raises the risk of GML. Therefore, the present findings reveal that HAPC-induced GML inspires the protection responses by up-regulating APOA4 and APOC3, and down-regulating GIF. These results may offer the basic information for the treatment of HAPC-induced gastric lesion in the future.

## Introduction

High-altitude polycythemia (HAPC) is characterized by excessive erythrocytosis (females, Hb ≥19 g/dL; males, Hb≥21 g/dL) and exists in 18% of the population residing at the Tibetan Plateau[1]. More red blood cells (RBCs) enable the lungs to obtain enough oxygen[2]. An increase of RBCs, stabilizes at a certain level in most people during long-term exposure to high altitudes; however, continuous increase of RBCs will result in serious clinical symptoms.

Gastric mucosal lesion (GML) is the most concomitant disease followed by HAPC and difficult to deal with. A GML has some defects in the gastric system that extends from the mucosa to the submucosa. A previous paper has reported endoscopic examinations in 98 GML patients from Lhasa, showing 29 patients with superficial gastritis, 26 patients with gastric ulcer and 3 patients with gastritis[3, 4]. Some researchers examined 5 GML patients in Madou county (4300 m, Qinghai province, China), having diffuse bleeding and erosion, as well as ulcerous necrosis in their stomachs[5]. There is an accumulating body of evidence that blood flowing in the micro-vessels (capillaries, arterioles and collecting venues) is crucial for the maintenance of the structure and functions of all tissues, including the gastrointestinal mucosa[6, 7]. In some situations, the gastric microcirculation is bypassed by arteriovenous shunting, which leads to severe gastric mucosal injury[8]. These results indicate that HAPC-induced GML may be associated with gastric mucosal ischemia caused by microvascular thrombosis due to excessive polycythemia. In addition, under normal conditions, a physiological balance exists between peptic acid secretion and gastroduodenal mucosal defense. Mucosal injury and subsequent peptic ulcers will occur when the balance between the aggressive factors and the defensive mechanisms is disrupted. The decrease in defense can be caused by many factors, including hypoxia[9]. Hypoxia is a primary cause for pathophysiological changes at high altitude. The hypoxia will result in decreased blood flow to the gastric mucosa, leading to ischemia and then subsequent destruction of the mucosal lining. Recent work uses an animal model of hypoxia-ischemia to induce gastric mucosal lesions, showing that hypoxia increases the expression of HSP-70, which will be activated for protecting cells from further being damaged[10]. However, little work has been done for the effect of ischemia and hypoxia on HAPC-induced GML patients at high altitude and the underlying molecular mechanisms remain unclear.

To control HAPC-induced GML, it is necessary to understand the molecular mechanism involved in the disorder. In this study, we compared the global gene expression of gastric mucosa from GML patients and healthy subjects to identify candidate molecules involved in HAPC-induced GML.

## Results

### Endoscopic imaging of the upper gastrointestinal tract

**Fig 1:**
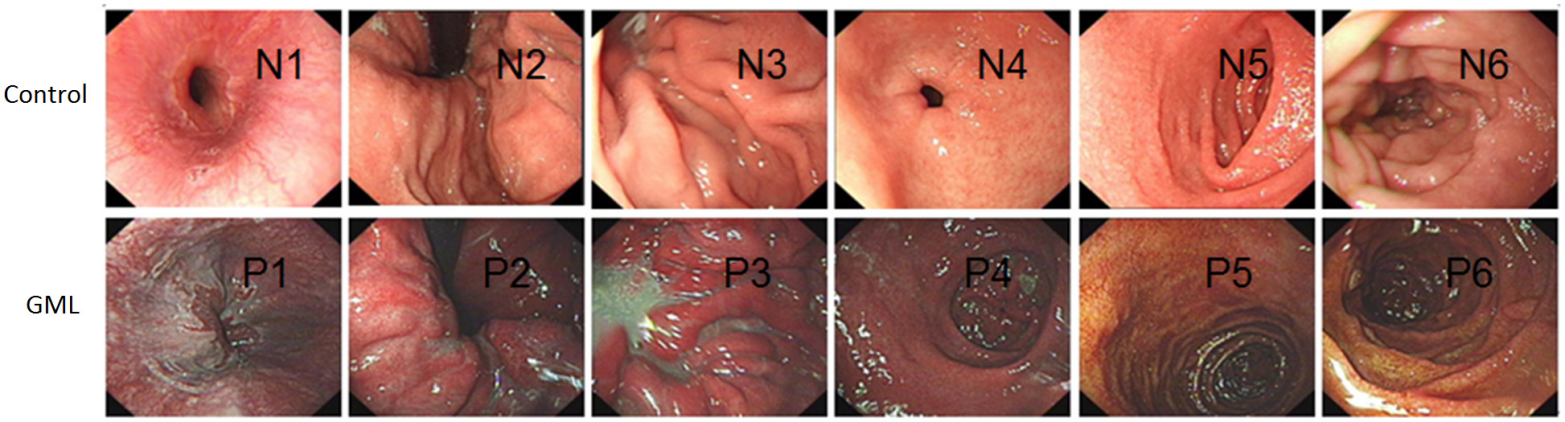
Endoscopic examination of the upper gastrointestinal tract. The upper gastrointestinal tract includes esophagus (N1/P1), cardia(N2/P2), gastric fundus(N3/P3), gastric antrum and body(N4/P4), the duodenal bulb (N5/P5) and descending portion(N6/P6). N stands for normal healthy subjects. P stands for polycythemia patients.

In the HAPC-induced GML group, the patients had evident mucosal hyperemia and edema compared with healthy controls. Furthermore, compared with that from healthy controls, endoscopic exam showed a darker color in the upper gastrointestinal tract in GML patients, including esophagus (N1/P1), cardia(N2/P2), gastric fundus(N3/P3), gastric antrum and body(N4/P4), the duodenal bulb (N5/P5) and descending portion(N6/P6) (86.3% vs 4.2%, p<0.001) (Fig 1). Moreover, in HAPC-induced GML group, the mucosa of patients was thin and red, and a fine meshwork of vessels could be observed under the mucosa as cord-like and branchlike structures. Furthermore, congestion was detected in areas where veins were slightly wider in GML patients when compared with healthy controls. In addition, due to the significant pink of the esophageal mucosa, the boundaries between esophageal mucosa and gastric mucosa could not be distinguished in GML patients. In contrast, the boundaries could be observed in healthy controls.

In HAPC-induced GML group, the color of the gastric mucosa of patients was darker, mainly manifested as a dark red, red-purple. Diffuse hyperemia and edema, as well as obvious changes of congestion, were observed. Also, in the descending segment of the duodenal bulb in HAPC-induced GML group, the mucosal color of patients was significantly browner and resembled a brownish red compared to that in the control group. In addition, the villi of the duodenal bulb were slightly enlarged with obvious hyperemia and swelling (Fig 1).

Abnormal contraction and relaxation only occurred in about 12.5% subjects, and most pyloric antra contracted and relaxed normally in the control group (*P* < 0.01). Additionally, in HAPC-induced GML group, bile was observed in both the fundus and body of the stomach of patients, as well as in the pyloric antrum and varying degrees of bile reflux was detected in nearly 86.4% patients. In contrast, gastric bile was observed in only 16.7% subjects in the control group (*P* < 0.001).

### Histopathological changes

**Fig 2:**
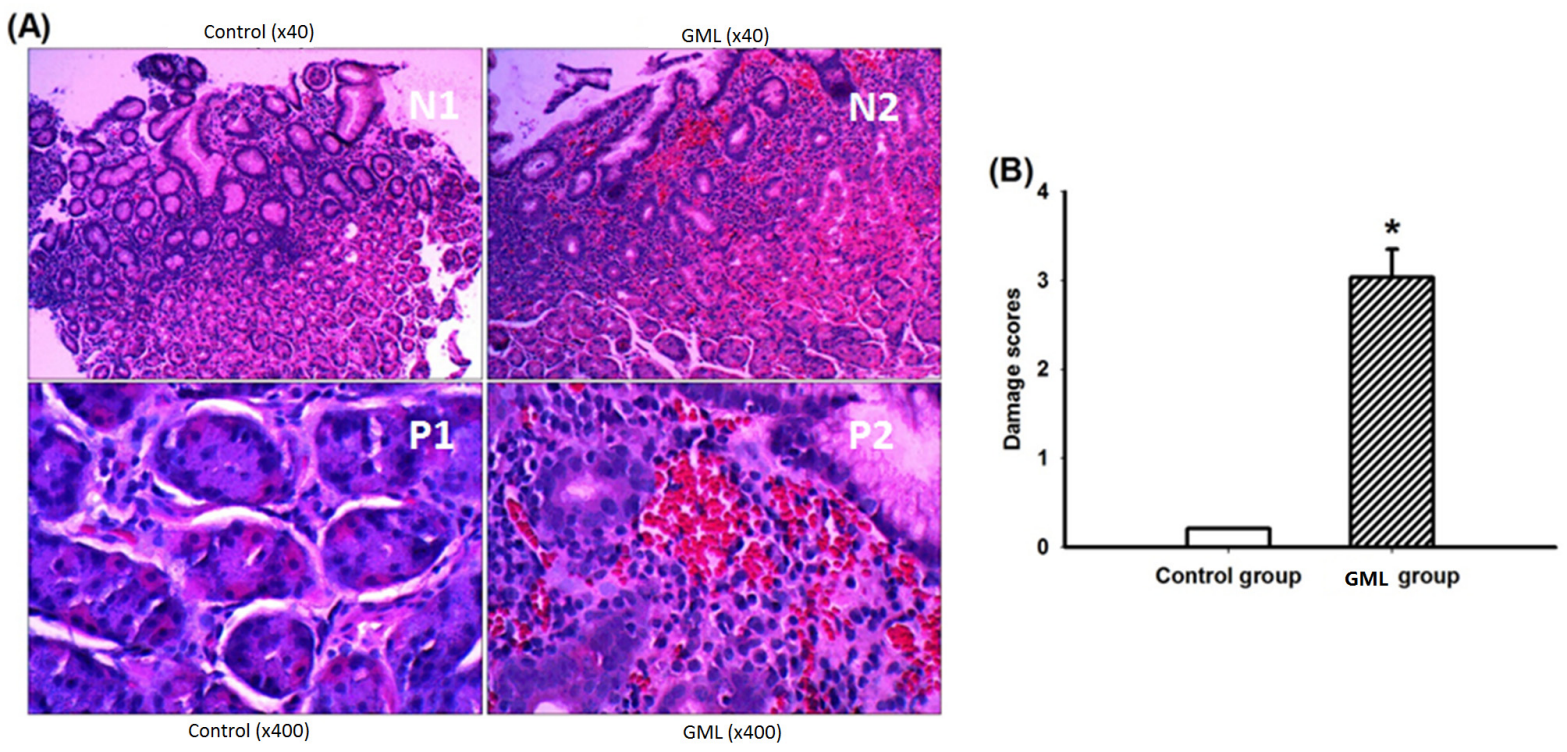
Histopathological examination of gastric mucosa sections. A, Hematoxylin-eosin stained results showed appearance of the gastric mucosa in antrum region of the control group and HAPC-induced GML group (× 40 and × 400); B, The gastric mucosal damage score in control and HAPC-induced GML group. The mean scores were significantly higher in the HAPC-induced GML group when compared with a control group (P < 0.01, n = 3 per group).

The typical characters of the normal gastric mucosa were shown in Fig 2A. Histopathological analysis revealed that HAPC induced severe congestion, edema and multiple hemorrhagic erosions in the gastric antrum mucosal tissues (Fig 2A). In HAPC-induced GML group, the mean score of gastric mucosal damage was 3.04 ± 0.312, and no significant damage was observed in the gastric mucosa in a control group (*P* < 0.01) (Fig 2B).

Under a low-magnification microscope, various changes in the vessels within the gastric mucosa were observed in HAPC-induced GML group, such as dilation and distortion accompanied by hyperemia and bleeding, which was statistically different from the control group (90.9% vs 8.3%, *P* < 0.001). Under a high-magnification microscope, the number of vessels per high power field was statistically significantly different in sections of the gastric mucosa between GML and control groups (24.68 ± 4.38 vs. 11.79 ± 2.43, *P* < 0.001). The statistical analysis for average vessel diameter showed that, the average vessel diameter per high power field in the sections of the gastric mucosa was significant higher in HAPC-induced GML group than in the control group (3.92 ± 1.15 vs. 1.59 ± 0.45, *P* < 0.001). Statistical analysis for RBC counts showed that RBC counts in sections of the gastric mucosa were higher in HAPC-induced GML group than in the control group (160.91 ± 62.53 vs. 30.33 ± 15.98, *P* < 0.001) (Fig 2).

### Hierarchical cluster analysis of differentially expressed genes (DEGs) in the gastric mucosa of GML patients

**Fig 3:**
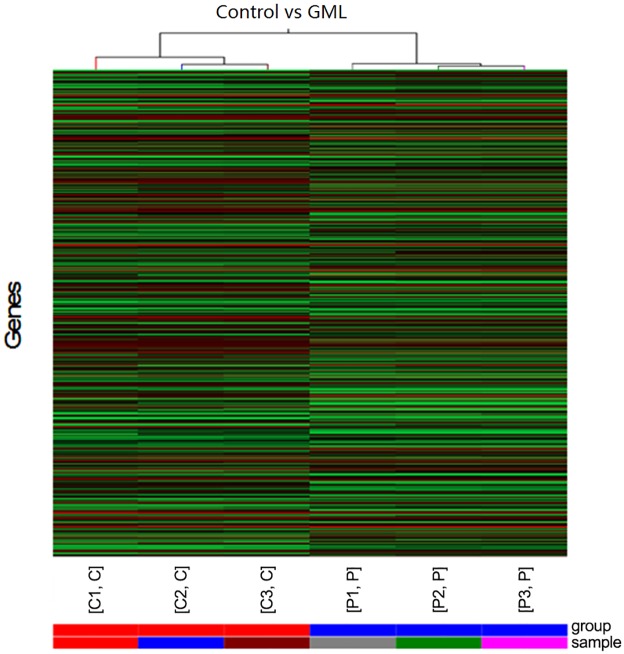
Hierarchical cluster analysis of the altered genes in the gastric mucosa of GML patients. The color code in each heat map was linear with green as the lowest and red as the highest. The increased genes expression was shown in green to red, whereas the decreased genes expression was shown from red to green.

We analyzed the spectrum of the DEGs in the gastric mucosa of GML patients and healthy controls within the context of the 6 mRNA-expression subtypes (Fig 3). Many DEGs showed mRNA-subtype-specific and clinical-subtype-specific patterns. The DEGs were considerably more diverse and recurrent within HAPC and control groups; however, the overall up-regulated DEGs were high in a control group and low in a HAPC-induced GML group.

Hierarchical cluster analysis of DEGs showed 4341 up-regulated genes and 5363 down-regulated genes in the gastric mucosa of GML patients. The levels of APOA4, APOC3, ADH4, ZG16, CHP2, CA1, BTNL3, PRAC, GCG, SPINK4, MLN, SLC26A3, HOXB13, RBP2, SI, CLCA1, FABP1, C19orf69 and BX641099 were markedly increased (Table 1), and the levels of GIF, ATP4B, PGC, ATP4A, AGXT2L1, TCN1, SLC9A4, PDILT, LTF, TMEM184A, TRIM50, PGA3, CKMT2, ALDH3A1, HSP90AA1, KCNJ16, BCL2L11, CLIC6, PGC, and CCKBR were dramatically decreased (Table 2). In particular, apolipoprotein genes APOA4 and APOC3 were 1473-fold and 1468-fold up-regulated (Table 1). In contrast, gastric intrinsic factor (GIF) was 1102-fold down-regulated (Table 2).

**Table 1:** Highly up-regulated genes in GML patients in comparison with controls.

GenBank	Gene symbol	Fold change	Description
**NM_000482**	APOA4	1473.33	apolipoprotein A-IV
**NM_000040**	APOC3	1468.55	apolipoprotein C-III
**NM_000670**	ADH4	1142.82	alcohol dehydrogenase 4,polypeptide
**NM_152338**	ZG16	709.82	zymogen granule protein 16 homolog (rat)
**NR_033977**	FLJ22763	692.39	uncharacterized LOC401081
**NM_022097**	CHP2	681.91	calcineurin B homologous protein 2
**NM_001738**	CA1	674.55	carbonic anhydrase I
**NM_197975**	BTNL3	645.10	butyrophilin-like 3
**NM_032391**	PRAC	550.04	prostate cancer susceptibility candidate
**NM_002054**	GCG	544.89	glucagon
**NM_04471**	SPINK4	427.95	serine peptidase inhibitor
**NM_000482**	MLN	427.32	motilin
**NM_000111**	SLC26A3	390.39	solute carrier family 26 member 3
**NM_006361**	HOXB13	379.02	homeobox B13
**NM_004164**	RBP2	301.37	retinol binding protein 2
**NM_001041**	SI	298.18	sucrase-isomaltase (alpha-glucosidase)
**NM_001285**	CLCA1	230.45	chloride channel accessory 1
**NM_001443**	FABP1	218.83	fatty acid binding protein 1
**NM_001130514**	C19orf69	166.88	chromosome 19 open reading frame 69
**BX641099**	BX641099	156.08	Fatty acid binding protein 1, liver [Source:HGNC Symbol;Acc:3555] [ENST00000495375]
**NM_001011552**	SLC9A4	119.65	solute carrier family 9 (sodium/hydrogen exchanger), member 4

**Table 2:** Highly TOP down-regulated genes in GML patients in comparison with controls.

GenBank	Gene symbol	Fold change	Description
**NM_005142**	GIF	1102.62	gastric intrinsic factor
**NM_000705**	ATP4B	778.50	ATPase, H+/K+ exchanging, beta polypeptide
**NM_001166424**	PGC	557.68	progastricsin
**NM_000704**	ATP4A	329.33	ATPase, H+/K+ exchanging, alpha polypeptide6 homolog (rat)
**NM_031279**	AGXT2L1	209.08	alanine-glyoxylate aminotransferase 2-like 1
**NM_001062**	TCN1	169.71	transcobalamin I
**NM_174924**	PDILT	105.87	protein disulfide isomerase-like, testis expressed
**NM_002343**	LTF	98.50	Lactotransferrin candidate
**NM_001097620**	TMEM184A	96.93	transmembrane protein 184A
**NM_178125**	TRIM50	95.69	tripartite motif containing 50
**NM_001079807**	PGA3	93.33	pepsinogen 3
**NM_001825**	CKMT2	89.52	creatine kinase, mitochondrial 2 (sarcomeric) member 3
**NM_000691**	ALDH3A1	85.57	aldehyde dehydrogenase 3 family, member A1
**NM_001017963**	HSP90AA1	84.59	heat shock protein 90kDa alpha (cytosolic), class A member 1
**NM_170741**	KCNJ16	83.65	potassium inwardly-rectifying channel, subfamily J, member 16(alpha-glucosidase)
**NM_207002**	BCL2L11	71.07	BCL2-like 11
**NM_053277**	CLIC6	70.38	chloride intracellular channel 6
**NM_176875**	CCKBR	67.16	cholecystokinin B receptor frame 69
**NM_000064**	C3	64.06	complement component 3 33receptor

### qRT-PCR analysis

**Fig 4:**
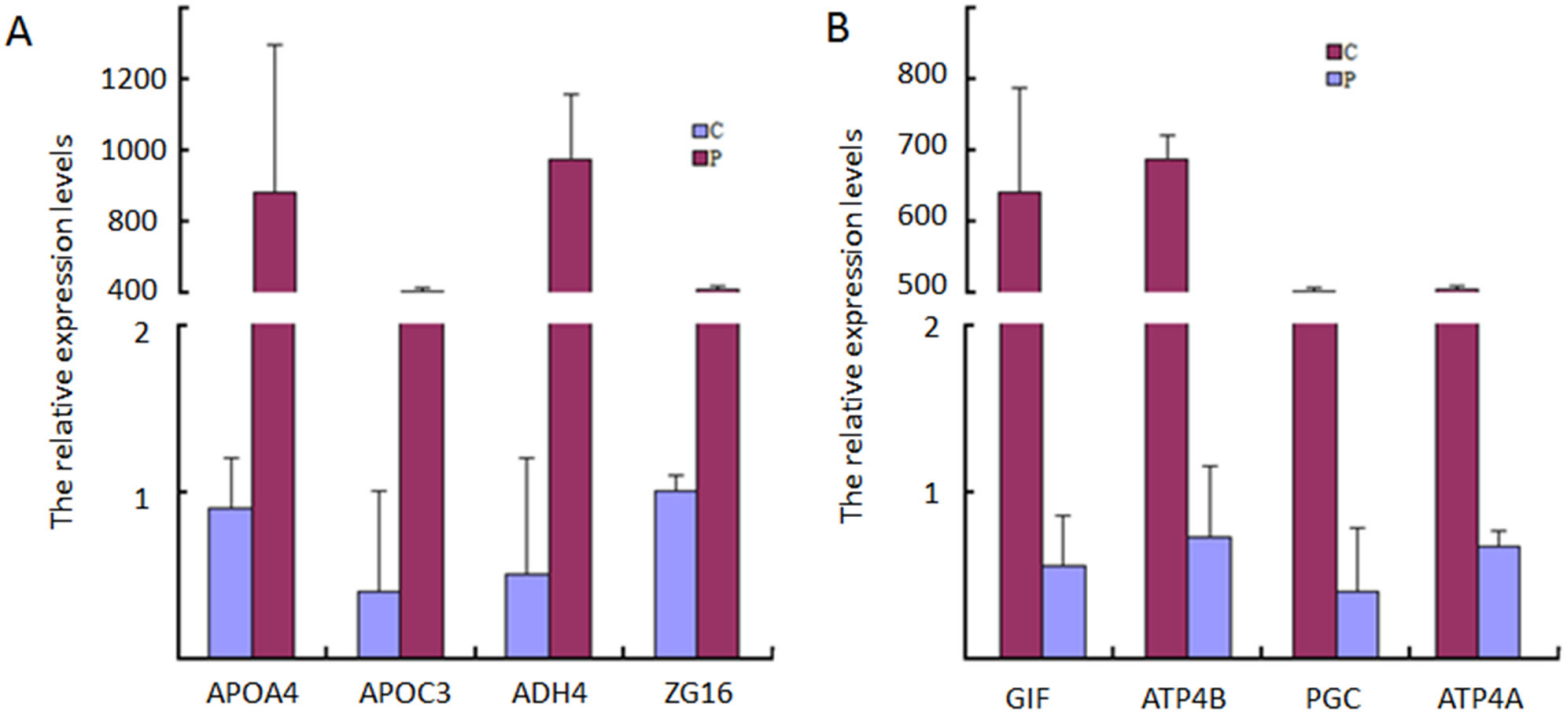
Validating microarray results using qRT-PCR. A, the top-4 up-regulated DEGs. B, the top-4 down-regulated DEGs. The results represented quantification of mRNA levels relative to β-actin. Normalized expression values were obtained by qRT-PCR (n = 3). C = Controls, P = GML patients.

To confirm the chip results, four TOP up-regulated genes were chosen for qRT-PCR analysis. The results showed that APOA4, APOC3, ADH4, ZG16 and CHP2 expression patterns were similar to that observed in the microarray experiments. Meanwhile, four TOP down-regulated genes including GIF, ATP4B and PGC were repeatedly identified by qRT-PCR (Fig 4). By qRT-PCR analysis of these genes in 3 pairs of tissues from healthy controls and HAPC-induced GML patients, APOA4 and APOC3 were more than 800-fold upregulated and GIF was 600-fold down-regulated in gastric mucosa tissues of HAPC-induced GML patients compared with healthy controls.

### Biological function analysis of DEG

**Fig 5:**
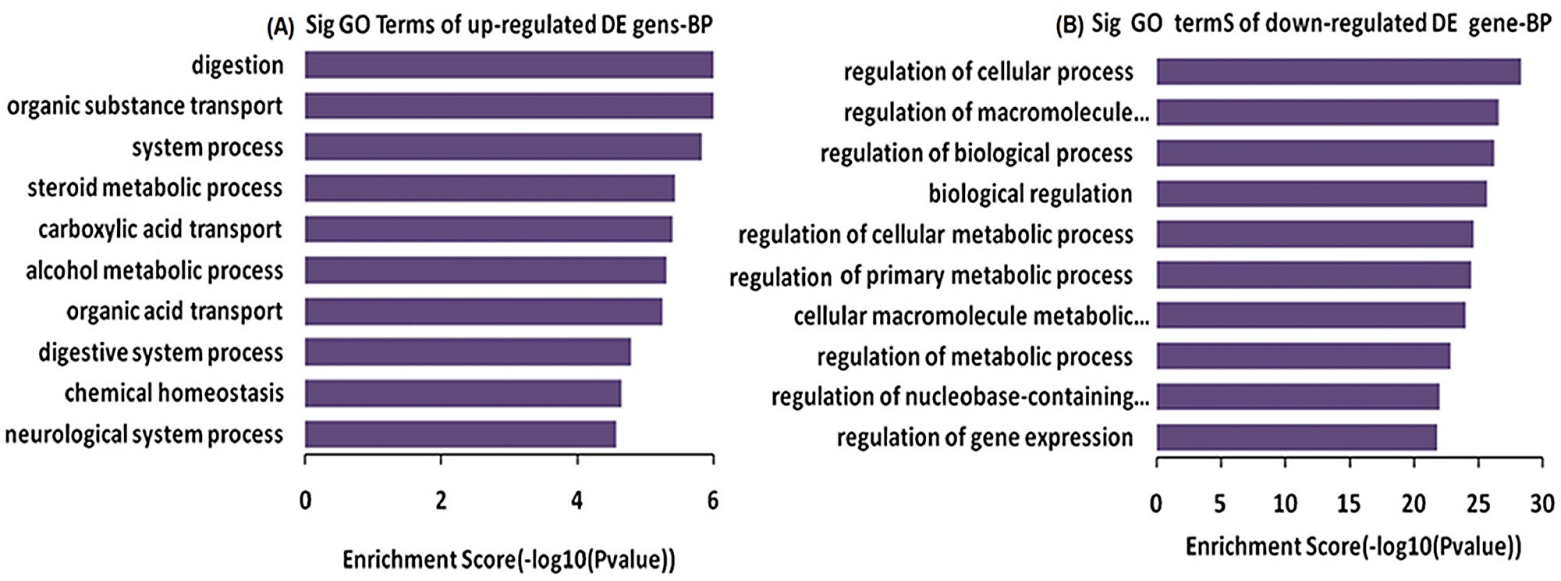
Gene ontology (GO) analysis used for analysis of the altered genes. A, The bar plot showed the top ten up-regulated Enrichment Score values of the significant enrichment; B, The bar plot showed the top ten down-regulated Enrichment Score values of the significant enrichment BP.

The identified DEGs were annotated in the GO format for biological function classification and in the pathway analysis format for elucidating the whole events in gastric mucosa tissues of GML patients compared with controls. In the GO analysis, the up-regulated DEGs involved in the digestion system, organic substance transport, carboxylic acid transport, and steroid metabolism and alcohol metabolism so on (Fig 5A); the down-regulated DEGs involved in the cellular metabolic process, macromolecule metabolic process, regulation of biological process, regulation of cellular metabolic process and macromolecule metabolism so on(Fig 5B).

**Fig 6:**
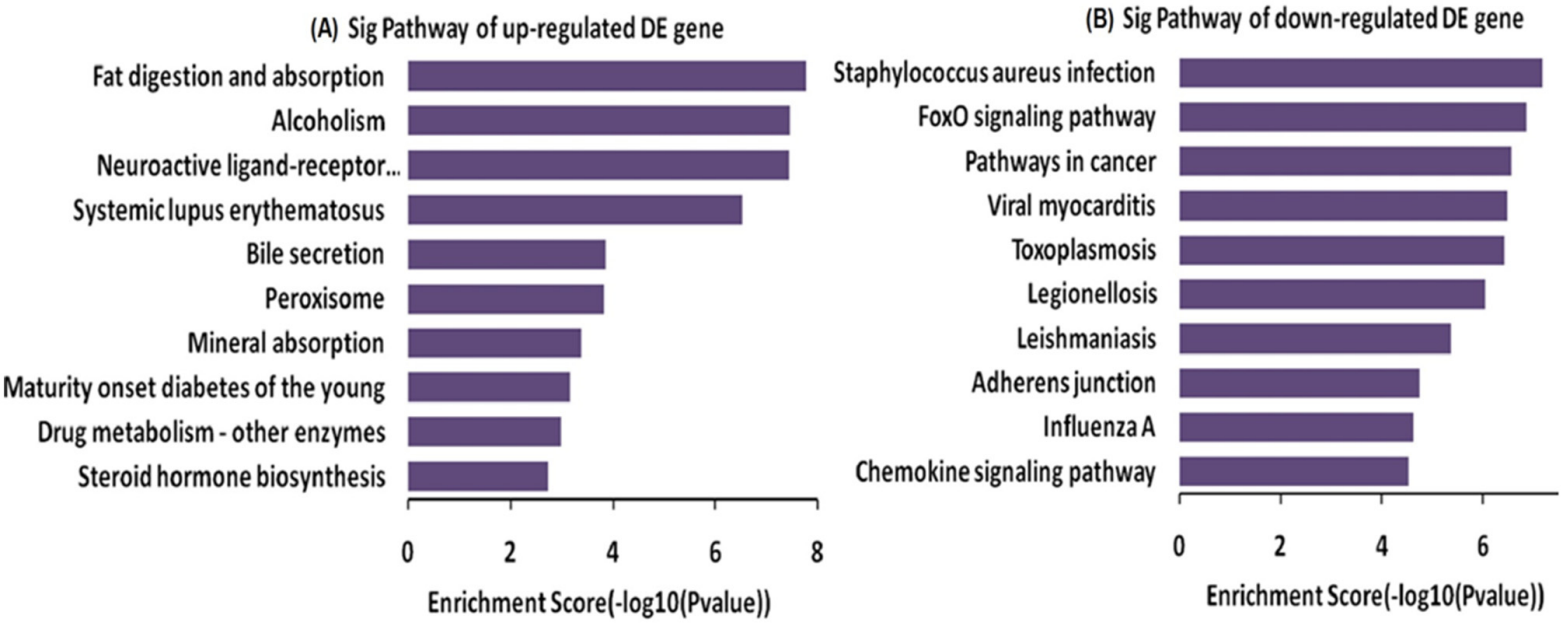
Pathway analysis of DEG. A: The bar plot showed the top ten up-regulated Enrichment Score values of the significant enrichment pathway; B: The bar plot showed the top ten down-regulated Enrichment Score value of the significant enrichment pathway.

KEGG pathway analysis revealed that the activities increased for digestion and absorption, including fat digestion and absorption, alcohol use, systemic lupus erythematosus, bile secretion, mineral absorption and peroxisome (which is essential organelle for redox signaling and lipid homeostasis) in the gastric mucosa tissues of GML patients compared with healthy controls (Fig 6A). Comparatively, the other pathways were down-regulated for immune activities in GML patients, including staphylococcus aureus infection, FoxO signaling pathway, cancer pathways, viral myocarditis and toxoplasmosis so on (Fig 6B).

### Clustering of DEGs in Chromosomal Domains

**Fig 7:**
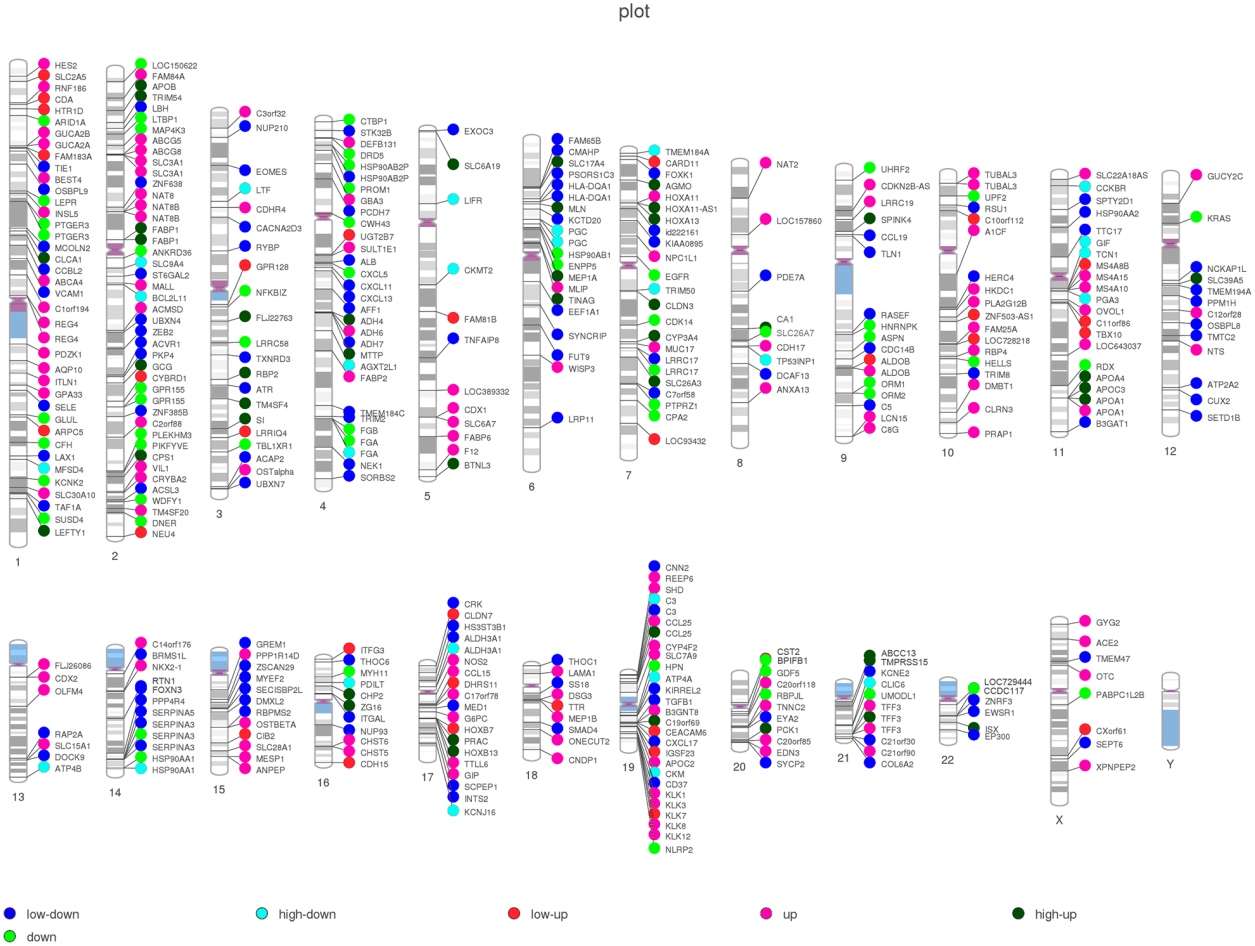
Whole chromosome view of expression levels of the 400 top DEGs mapped to 23 chromosomes. Each circle represented one DEG. Expression levels were normalized to six grades (Low-up /4-6 fold, up/7-10 fold, high up/more than 10 fold; Low-down /4-6 fold, down/7-10fold, high down/more than 10 fold).

Finally, the top-400 DEGs were marked on 23 chromosomes (Fig 7). There were no top DEGs on 13p, 14p, 15p, 20p, 21p, 22p and Chromosome Y, which had fewer quantitative-trait loci for lipid metabolism. Most top-DEGs were clustered on chromosomes 1, 2, 4, 6, 7, 11, 19 and 21. The top-3 DEGs (fold change more than 1000) were all located on the long arm of Chromosome 11, including APOA4 (chr11:116691770–116691711), APOC3 (chr11:116703530–116703589) and GIF (chr11:59603362–59603303) genes.

## Discussion

The Qinghai-Tibetan Plateau is the largest and highest plateau, which holds the largest high-plateau population in the world. Due to an abnormal increase in RBC, there is increasing blood viscosity and both microcirculation disturbances and systemic disorders in the population. However, no effective prevention and control measures have yet been utilized. Generally, the incidence of HAPC increases with the elevation of altitude. However, because of varieties of living environments such as altitude, and diverse ethnic groups, the incidence of HAPC is quite different around the world[11].

The pathogenesis of HAPC is complex. It has been widely reported that increased synthesis and release of erythropoietin, induced by long-term exposure to high-altitude hypoxic conditions, which is a key factor for HAPC development [12]. Here, Hb concentration, RBC counts, and hematocrit (HCT) were significantly higher in HAPC-induced GML group than in a control group. This was also consistent with the notion that the initiating factor of HAPC was the high-altitude hypoxia-induced enhancement of bone marrow erythropoiesis, which induced RBC hyperplasia and related clinical manifestations. There was no significant difference in white blood cell (WBC) counts detected between the two groups, suggesting that high-altitude hypoxia might have more subtle effects on bone marrow leukocytes. However, it was found that platelet (PLT) counts were lower in HAPC-induced GML group than in a control group. It remains unclear if this observation is related to the decline in platelet regeneration caused by high-plateau hypoxia. In addition, the oxygen saturation of arterial blood was significantly lower in HAPC-induced GML group than in a control group, which was consistent with previous reports[13, 14], showing that this might be associated with excessive RBC hyperplasia as well as increased blood viscosity and flow resistance in GML patients.

Previous work showed that in the presence of high-altitude hypoxia, the dynamic equilibrium between in vivo nitric oxide and endothelin was broken, causing enhanced vasoconstriction and increased systemic peripheral resistance. This led to the dilation of mucosal vessels. Meanwhile, high-altitude hypoxia-induced HAPC caused increased blood viscosity, slow blood flow, and severe local mucosal congestion, resulting in severe vascular hyperemia and even rupture. As a consequence, there was microcirculation disturbance of the gastric mucosa[15]. Considering the environment contributing to long-term high-altitude hypoxia, increasing RBC can cause the damage of the gastrointestinal mucosa and compromise normal physiological functions such as digestion and absorption.

The Whole Human Genome Oligo Microarray is a broad view that represents all known genes and transcripts in the human genome. Sequences are compiled from a broad source survey, and then verified and optimized by aligning the human genome. In this study, gastric mucosal tissues samples from GML patients and controls were analyzed using genome microarrays, in which 4941 genes (fold change ≥2) were up-regulated and 5363 genes (fold change ≤0.5) were down-regulated in the patients with GML. Using GO and pathway analyses, the functions of DEGs were explored. The results indicated that HAPC-induced GML was a process involving multiple genes and pathways.

The microarray assay showed that apolipoprotein A-IV (*APOA4*) was a strong up-regulated gene (fold change = 1473.33) in GML patients compared with the healthy subjects. As reported in previous studies, *APOA4* is associated with circulating high-density lipoproteins and plays a role in cholesterol transport and lipid metabolism[16]. Exogenous administration of *APOA4* acts peripherally as well as centrally to suppress food intake. Peripheral administration of exogenous *APOA4* inhibits food intake and increases neuronal activation in the NTS, and these are mediated via a CCK-dependent pathway [13]. Therefore, HAPC-induced GML probably inhibited food intake caused by *APOA4* overexpression. In addition, other report describes a new anti-inflammatory property of *APOA4*, the overexpression of *APOA4* in an apoE-deficient mice significantly reduces the progression of atherosclerosis and proinflammatory cytokines induced by LPS, when compared with apoE-deficient littermates^18^. So *APOA4* also probably plays an important role in protection of the gastric inflammation.

APOC3, in the same family with APOA4, inhibits triglyceride hydrolysis. The common genetic variant of APOC3 contributes to metabolic syndrome in adults[17]. APOC3 plays an important role in regulating the metabolism of triglyceride-rich lipoproteins and has been regarded as a main component of chylomicrons and very-low-density lipoprotein (VLDL). The APOC3 gene is located on chromosome 11q and often changes. The SstI polymorphism on the 3' untranslated region (UTR) of the APOC3 gene is linked with an increase in the concentrations of triglycerides and total cholesterol (TC)[18].

Meanwhile, the third member of APOA family APOA1, is also highly up-regulated (more than 100 fold). APOA1 is the main component of high-density lipoprotein (HDL) and participates in the process of cholesterol esterification as a cofactor for the acyltransferase of lecithin-cholesterol. Thus, APOA1 becomes very important in cholesterol efflux from peripheral cells. The APOA1 gene also involves in the metabolism of lipids [19].

From above information, it is easy to find that APOA family mainly involves in lipid metabolism. Furthermore, the genes coding for apolipoproteins APOA1, APOC3 and APOA4 are closely linked and tandem locate within a 15- kb DNA segment on the long arm of chromosome 11[20]. Thus, all these genes have synergistic functions and increase the levels of lipid, which is a potent inhibitor of gastric acid secretion[21]. Actually, APOA4 is regarded as a kind of inhibitor of gastric acid secretion [22]. For another member of APOA family, APOC3 inhibits lipoprotein lipase and hepatic lipase; it is thought to inhibit hepatic uptake of triglyceride-rich particles [23]. As just mentioned, the high level of lipid may inhibit gastric acid secretion [24]. From these results, all the three members of APOA family may inhibit the gastric acid secretion and showed synergistic protecting functions for GML patients because gastric acid will increase the severity of gastric injury.

Microarray analysis showed that gastric intrinsic factor (GIF) was a highly down-regulated gene in GML patients compared with that from healthy controls (fold change = 1102.62). *GIF* involves in the uptake, transport, and storage of vitamin B12. Lots of evidence has suggested that vitamin B12 deficiency in the patients with gastric bypass or vertical banded gastroplasty is due to inadequate secretion of *GIF*[25]. Megaloblastic anemia and neurological disturbances are common symptoms because of the deficiency of the vitamin B12. Therefore, GIF deficiency will cause acute anemia, which may be the main protecting mechanism for GML by antagonizing HAPC.

Other possible mechanisms were also explored here. The chip results showed that three bioelectricity related genes (*ATP4B*, *ATP4A and PGC*) were extremely down-regulated. The gastric H, K-ATPase comprises two subunits: a catalytic α subunit and a β subunit. The gastric H^+^, K^+^-ATPase is an ATP-driven proton pump responsible for generating a million-fold proton gradient across the gastric membrane[15], which pumps gastric acid in cytoplasmic tubular membranes and then in the microvilli of the expanded secretory canaliculus in the stimulated parietal cells. The gastric H, K-ATPase moves from the tubule vesicles to the apical membrane in the canaliculus of the stimulated state and secretes gastric acid by an electroneutral, ATP-dependent hydrogen-potassium exchange[26], which indicates that HAPC-induced GML may affect H,K-ATPase activity and decrease gastric acid secretion. In addition, ATPase enzymes are the marker for the cellular integrity and hence decreasing ATPase activity indicates defects in cellular integrity. A previous report shows that diabetes cause significant reduction in the cellular ATPase enzymes levels as compared to normal rats[27]. Here the decreasing H, K-ATPase activity made the gastric mucosal surface more fragile and susceptible to hypoxia for GML patients.

Certainly, there are some limitations for present study. Firstly, 6 participants was a small population to explore the molecular mechanism of GML. The validation of the APOA4, APOC3, and GIF expression was only performed in the 3 patients and 3 healthy controls. The conclusion of the important role of APOA4, APOC3, and GIF in HAPC-induced GML should be validated in a larger cohort. The conclusion based on the data seemed arbitrary. To avoid such whimsicality, qRT-PCR analysis for the three genes were conducted in other 12 GML patients and 14 controls in the subsequent work. The similar results were still obtained: APOA4 and APOC3 were more than 800-fold upregulated and GIF was 600-fold down-regulated in the tissues of HAPC-induced GML patients compared with healthy controls. Secondly, the more detail molecular mechanisms were still needed to be explored. The pathogenesis HAPC-induced GML was not explored in the paper although which was our initial aim. The disease can be elucidated in vitro using gastric cells from GML-related iPS cells[28]. Finally, APOA4 and APOC3 may have different functions, which were not studied here.

In any way, the results of this study were completely from the whole genome microarray data comparing GML patients and controls, providing clues to the molecular protecting mechanism for HAPC-induced GML. APOA4 and APOC3 genes originated from a common ancestor and may act as a humoral inhibitor of gastric acid secretion while gastric acid promotes ulceration formation. GIF deficiency activates a program of acute anemia, which may antagonizes polycythemia while polycythemia raises the risk of GML. Therefore, the present findings revealed that HAPC-induced GML inspired the protection responses by significantly up-regulating the levels of APOA4 and APOC3, and down-regulating the levels of GIF. These results may offer some basic information for the treatment of gastric lesion caused by HAPC in the future.

## Methods

### Patients

The experimental protocol was established according to the ethical guidelines of the Helsinki Declaration and approved by the ethics committee of People's Hospital of Tibet Autonomous Region, Lasha, China. Written informed consent was obtained from the individual participants. At June 2014, 3 patients, who lived on the Tibetan Plateau at an average altitude of 3600 to 4800 m, with a definite diagnosis of high altitude polycythemia (HAPC) were enrolled in a HAPC-induced GML group, and all had symptoms as observed by gastroscopy. Meanwhile, 3 healthy Tibetans, who had screening requirements for gastrointestinal endoscopic examination during the corresponding period, were considered as a control group. All subjects were from Lhasa, Nagqu, and Rigaze of Tibet and all of them were indigenous and had lived in their regions for at least 30 years. Each patient was matched to a control according to gender, birthplace, age, lifestyle, diet, BMI, height of location and work intensity. Body mass index and per capita income are main factors affecting gastric injury caused by inflammation [29], so these factors were carefully considered. All the subjects were native male Tibetans from Lhasa within 40–45 years of age. All the subjects were asked to sign an informed consent term form before their tissues were obtained.

Before endoscopy, peripheral venous blood was sampled for a routine test. Pulse oximetry was used to measure the oxygen saturation of arterial blood. Inclusion criteria for the study were performed according the definition of HAPC. HAPC was defined by the 2004 Qinghai International High Altitude Medicine Conference, including a hemoglobin (Hb) concentration > 21 g/dl (male) and > 19 g/dl (female)[1]. Exclusion criteria were: chronic pulmonary diseases including emphysema, bronchitis, bronchiectasis, alveolar fibrosis, lung cancer and other serious pulmonary diseases; chronic respiratory disorders or secondary polycythemia due to hypoxemia caused by certain chronic diseases; severe diseases of the heart, brain, lungs, liver, kidneys, endocrine system and hematopoietic system; alcohol abuse, drug addiction, poor mental health or other conditions inappropriate for gastroscopy; pregnant or lactating women; obstructed gastrointestinal tract, and medical histories such as recent gastrointestinal bleeding. The diagnosis of chronic gastritis was made according to the Chinese Consensus on Chronic Gastritis, formulated in Shanghai in 2006. The histopathological diagnosis was based on the Operative Link on Gastritis Assessment (OLGA) staging system[30].

### Endoscopic detection

A rigorous endoscopic surveillance was maintained for all subjects [31]. Ten mL of viscous lidocaine hydrochloride Mucilage (Jiangsu Jichuan Pharmaceutical Co., Ltd., China) was orally administered to study subjects for HAPC assessment. A gastroscope (OLYMPUS GIF-260) was used for examination. All of the subjects were examined using the same endoscope by an experienced endoscopist. The strength and type of endoscopic lamp light source were consistent throughout the study. The color of esophageal, gastric and duodenal mucosal lesions was carefully assessed, as well as contraction and relaxation of the pyloric antrum. The color changes of the upper gastrointestinal mucosa for patients with HAPC were observed by endoscopy. The photos were taken for the upper gastrointestinal tract, including the esophagus, cardia, gastric fundus, gastric antrum and body, the duodenal bulb and the descending portion. Endoscopic mucosal biopsy was performed in all subjects.

### Histological analysis

The mucosa was collected at the Endoscopy Surgery Department of People’s Hospital of the Tibet autonomous region (Tibet, China). In total, we analyzed 12 gastric antrum biopsies, in which 6 samples were from GML patients and other 6 samples from healthy controls. The samples were snap-frozen in liquid nitrogen immediately after surgical excision and stored at -80°C until use. Gastric mucosal tissues were fixed in formalin and embedded in paraffin for histological analyses. Five-μm sections were stained with hematoxylin and eosin (Sigma). The results were evaluated by two researchers in a blinded fashion. The mucosa were considered injured if one or more of the following criteria were met: discontinuous surface, dilated gland, hemorrhage, or superficial cells damaged [32].

### RNA extraction

Gastric mucosal samples from 3 GML patients and 3 controls were pooled for each genotype. Each pool was placed in 3 mL Trizol reagent (Invitrogen Corporation, Carlsbad, CA, USA) and homogenized with a homogenizer. After extraction with chloroform, RNA was precipitated with isopropanol. The resultant pellet was finally resuspended in TE buffer (10 mM Tris-HCl, pH 7.5, 1 mM EDTA). After DNase digestion, RNA quantification and purity was assessed at 260/280 nm using an Agilent-Bioanalyzer (Agilent, Palo Alto, CA, USA). RNA integrity and genome DNA contamination were measured via denaturing agarose gel electrophoresis.

### Microarray data analysis

Single-color gene-expression profiles were produced by comparing the expression-level difference between the experimental subjects and controls. These profiles were constructed by 4 × 44 K oligonucleotide microarrays from Agilent Technologies (Palo Alto, CA, USA). The mouse genome microarray covers about 41,000 genes and transcripts. The representative sequences were obtained from public databases, including RefSeq, Ensembl, Unigene, and UCSC Goldenpath. The RNA samples were amplified and labeled using the labeling kit from Agilent Technologies (Palo Alto, CA, USA) according to manufacturer instructions and hybridized with the Agilent mouse genome microarray in the hybridization chambers of Agilent’s SureHyb. After hybridization and washing, the hybridized slides were scanned by DNA microarray scanner (G2505B) and evaluated with the Agilent Feature Extraction Analytics software (version 9.5.3). The Feature Extraction placed microarray grids, rejected outlier pixels, determined feature intensities and ratios, flagged outlier pixels, and compiled QC reports. All the procedures were conducted by KangChen Bio-Tech, Shanghai, China. The Agilent GeneSpring GX software (version 7.3, Agilent Technologies, CA, USA) was used to calculate and record the signal intensities representing the gene expression levels generated by the Agilent 4 × 44 K One-Color Whole Genome Microarray. For inter-array normalization, a median normalization was used. For data analysis, fold changes were applied to select the differentially expressed genes (DEGs) using 2-fold change cutoff. Genes were regarded as up-regulated gene with 2-fold or higher expression compared to a control; genes were regarded as down-regulated genes with 2-fold lower expression, and 0.50- to 1.99-fold changes could be regarded as non-significant. Therefore, the profiling identified a subset of the total number of DEGs.

### Gene ontology (GO) and Kyoto encyclopedia of genes and genomes (KEGG) analysis

GO and KEGG databases were used to predict DEGs involved in biological functions and signaling pathways. The GO project provides a controlled vocabulary to describe gene and gene product attributing in any organism (http://www.geneontology.org). The ontology covers three domains: Biological Process (BP), Cellular Component (CC) and Molecular Function (MF). Fisher’s exact tests were performed to investigate if there were more overlaps between the DEGs list and the GO annotation list than expected by chance. The p-value denotes the significance of GO terms enrichment in the DEGs. The lower the p-value, the GO term is more significant (*P* < 0.05). Pathway analysis is a functional analysis mapping genes to KEGG pathways. The *P*-value denotes the significance of the pathway correlated to the conditions for EASE-score, Fisher and Hypergeometric *P* value). The lower *P*-value, more significant is for the pathway (The recommend *P*-value cut-off is 0.05).

### Real time quantitative reverse transcription polymerase chain reaction (qRT-PCR)

The reliability of chip results was confirmed by qRT-PCR analysis for top DEGs, including APOA4, APOC3, ADH4, ZG16, CHP2, GIF, ATP4B, PGC and Actin gene as a control. Total RNA was isolated from gastric mucosal samples using Trizol reagent (Invitrogen, USA) according to the Manufacturer’s instructions. Five micrograms of RNA were reverse-transcribed using a reverse transcriptase reaction kit (ABI biosystems, Foster City CA, USA). Platinum TaqDNA polymerase was purchased from ABI. Using the primers from Table 3, qRT-PCR was performed in triplicate using SYBR Green PCR Master Mix and reactions were carried out on 7500 Fast real-time PCR detection system (ABI biosystems, Foster City CA, USA) with the amplified conditions: 95°C for 10 min, 40 cycles of 95°C for 10s, 60°C for 34s and 60°C for 60s. The relative expression value was calculated by 2^-ΔΔT^ method.

**Table 3:** Primers and product size of genes that were tested with qRT-PCR.

Gene	Length	Forward primer	Reverse primer
**Actin**	**107 BP**	GGACCTGACTGACTACCTCAT	CGTAGCACAGCTTCTCCTTAAT
**APOA4**	**183 BP**	AAAGAGAGCCAGGACAAG	GGACAGACAGACAGACAG
**APOC3**	**160 BP**	TCAGAGGCCGAGGATGCCTC	AAGCCATCGGTCACCCAGCC
**ADH4**	**160 BP**	TGCAAGTTTTGTCTGAGTCC	CCAAAGAAATGGTAAACTGG
**ZG16**	**150 BP**	AGTTCCTCCTCACCTCTA	TGTAACCTTCTGATGGCTAA
**GIF**	**160 BP**	GCACCTTCCAGCCCCAACGC	GGAGAGGAGTTGGCCAGCAG
**ATP4B**	**108 BP**	GACCACCACCTACAAGAT	CTCAATGCTGATGAAGAACA
**PGC**	**160 BP**	GTGCCCCTGAAGAAATTTAA	GCCATGGGCTCGTAGGTCAC
**ATP4A**	**160 BP**	CGCCTGGCCAGTAAGAACTG	TGGTTGTCAAACCACAGATG

### Human Transcriptome Map

The chromosomal position of DEGs was established. We integrated these mapping data with genome-wide messenger RNA expression profiles as provided by microarray data. Four hundred top DEGs were assigned their chromosomal positions. The results of Human Transcriptome Map generated gene expression at specific region on 23 chromosomes. The map revealed a clustering of DEGs to specific chromosomal regions.

### Statistical analysis

Data were presented as the mean ± SD. Student’s t-test was used to calculate the statistical significance of paired data where appropriate. Statistical significance was defined as *P* < 0.05.
